# Individualizing Systemic Therapies in First Line Treatment and beyond for Advanced Renal Cell Carcinoma

**DOI:** 10.3390/cancers12123750

**Published:** 2020-12-13

**Authors:** Yasir Khan, Timothy D. Slattery, Lisa M. Pickering

**Affiliations:** The Royal Marsden Hospital NHS Foundation Trust, Fulham Road, London SW3 6JJ, UK; yasir.khan@rmh.nhs.uk (Y.K.); tim.slattery@rmh.nhs.uk (T.D.S.)

**Keywords:** renal cell cancer, immune checkpoint inhibitors, tyrosine kinase inhibitors, biomarkers, individualization

## Abstract

**Simple Summary:**

In recent years, a number of new, effective treatments have become available for advanced renal (kidney) cancer. However, with more drugs available it has become more difficult to decide which drugs to choose for particular patients, and in which order to use them. In addition, the new treatments do not work for all patients and they can have troublesome side effects. At the moment, these choices are made according to factors including the type of renal carcinoma from a biopsy, the extent of cancer for that patient, their previous health and their current fitness. The options are summarized in guidelines although these do not make recommendations for individual patients. It is hoped that ongoing research will uncover new tests that allow these decisions to be made more accurately in a “personalized” manner. This article describes how the process is undertaken at present and how it may change in the future.

**Abstract:**

Therapeutic options for treating advanced renal cell cancer (RCC) are rapidly evolving. Vascular endothelial growth factor (VEGF)-directed therapy, predominantly VEGF receptor (VEGFr) tyrosine kinase inhibitors (TKIs) had been the most effective first line treatment since 2005 irrespective of International Metastatic RCC Database Consortium (IMDC) risk stratification. However, immune checkpoint inhibitors (ICI) have recently changed the treatment paradigm for advanced RCC particularly as the first-line systemic treatment modality. The combination of Ipilimumab and Nivolumab provides better disease control and long-term outcomes compared with the anti-VEGFr TKI Sunitinib for IMDC intermediate- to poor-risk patients and we now have the option of using ICI with TKI upfront for all IMDC risk groups. This poses a challenge for physicians, both to select the most suitable first line regimen and the most suitable subsequent therapy given the lack of data about sequencing in this setting. This treatment landscape is expected to become more complex with the emerging treatment options. Moreover, these therapeutic options cannot be generalized as significant variability exists between individual’s disease biologies and their physiologies for handling treatment adverse effects. Notable efforts are being made to identify promising predictive biomarkers ranging from neo-antigen load to gene expression profiling. These biomarkers need prospective validation to justify their utility in clinical practice and in treatment decision making. This review article discusses various clinicopathological characteristics that should be carefully evaluated to help select appropriate treatment and discusses the current status of biomarker-based selection.

## 1. Introduction

Renal cell carcinoma (RCC) is the most common type of kidney cancer with increasing incidence worldwide in recent years [1]. The subtypes of RCC differ in their morphological and histological features as well as in the biology and molecular pathways that drive cancer growth. The most common subtype of RCC is clear cell RCC (ccRCC) which accounts for 75–85% of cases [2]. Other sub-types are sometimes collectively termed “non-clear cell” for convenience but are biologically and genetically distinct. These include papillary (15–20%), chromophobe (5%), collecting duct, medullary and translocation RCC [3,4]. Papillary RCC (PRCC) is further subdivided into type 1 and 2 PRCC based on certain genetic alterations.

RCC is one of the few solid organ malignancies in which it has long been recognized that the immune system plays a significant role. RCC does not carry a high mutation burden but nevertheless is responsive to immune modulation. Spontaneous regression is a well observed phenomenon in RCC and therefore, immune modulating agents like Interferon (IFN) and Interleukin-2 (IL-2) were commonly used treatments before the advent of TKIs [5,6,7]. Although durable, complete responses were seen with IL-2 in particular, that was achieved in fewer than 10% of patients and the effectiveness was further limited by a high rate of treatment-related toxicity.

Vascular endothelial growth factor (VEGF)-directed therapies, including anti-VEGF receptor (anti-VEGFr) TKIs became the new standard of care in 2005 following enhanced understanding of RCC biology. Their effectiveness is well established in ccRCC on the basis of randomized phase III registration studies [8,9,10,11]. These agents are also widely used in non-ccRCC on the basis of open label phase two trials, and reported off trial experience, but lack level one evidence. Over the last five years, immune checkpoint inhibitors (ICI) have further transformed the treatment of advanced RCC. These drugs work particularly well for cancers with increased mutational load and highly expressed neo-antigens. Nivolumab was the first anti-Programmed cell death (anti-PD-1) drug approved in the second line setting for advanced RCC in 2015 based on the results of the Checkmate 025 phase III trial [12]. Since then, ICI have been trialed in the first line setting and we now have a plethora of first line treatment options for individual IMDC risk categories ranging from ICI-based combinations and new generation TKIs which add to and now have largely superseded earlier generation TKIs. The updated treatment algorithms of European Society for Medical Oncology (ESMO) as well as National Comprehensive Cancer Network (NCCN) have incorporated all but the most recently presented of these data [13,14].

## 2. Selection of Treatment

**Figure 1:**
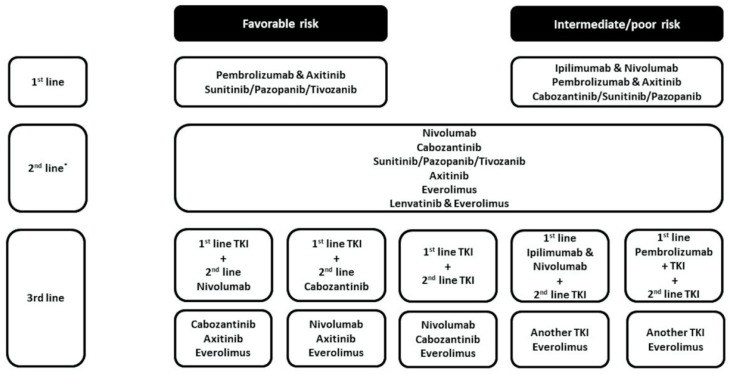
Treatment algorithm for International Metastatic Renal Cell Cancer Database Consortium (IMDC) risk categories [13]. * Based on 1st line treatment. TKI: Tyrosine kinase inhibitors.

As the number of approved systemic options to treat advanced RCC patients continues to increase, it has become increasingly challenging to select between them. Treatment algorithms provide general guidance (Figure 1) but data that clearly guide selection and sequencing at a personalized level in the first line setting and beyond are lacking. In this review article we will discuss how demographic, prognostic, clinical, and increasingly biomarker information can be used to individualize the treatment for the advanced RCC patients.

### 2.1. IMDC Risk-Based Treatment Selection

It has been recognized for some time that patients with advanced RCC can be categorized by clinical criteria into differing risk groups that have variable prognostic outcomes. The most commonly used model for risk assessment in current use is the International Metastatic RCC Database Consortium (IMDC) classification. In this model, patients are grouped as favorable (score 0), intermediate (score 1–2), and poor risk (score 3–6) based on their Karnofsky performance status, time from the diagnosis to initiate systemic treatment, hemoglobin level, neutrophil and platelet count and serum calcium level. Some reported clinical trials have utilized these risk criteria in defining the eligible trial population, and almost all current trials report efficacy outcomes stratified by IMDC risk category. Furthermore, the approvals of some treatments are restricted by risk category (Figure 1). Thus, the risk category of individual patients can help guide therapy choices.

There are now various management options to treat favorable risk patients including active surveillance, single agent TKI, or most recently the option of using ICI upfront in combination with a TKI. Anti-VEGFr TKI monotherapy has been the treatment of choice for treating advanced RCC patients with IMDC favorable risk disease since the mid 2000s. Both Sunitinib and Pazopanib are first generation multi-targeted TKIs and the efficacy benefits of each were seen across risk groups, including the favorable risk cohort. Tivozanib is a second generation TKI that was compared against Sorafenib as initial or second-line therapy for patients with advanced RCC in the TIVO-1 open label, randomized phase III trial. The majority of patients had favorable or intermediate risk disease. This trial showed a statistically significant PFS benefit for Tivozanib overall, with PFS of 16.7 months for Tivozanib versus 10.8 months for Sorafenib favorable risk patients (HR 0.59; CI 0.37–0.92; *p* = 0.018) [15]. Despite positive results for TIVO-1, Tivozanib has, as yet, failed to get regulatory approval from the Food and Drug Administration (FDA) but is being used in Europe and United Kingdom (UK) since its approval by the European Medical Agency (EMA) in 2017 [15].

The combinations of Pembrolizumab with Axitinib, and Avelumab with Axitinib, have recently been added to the list of FDA and EMA-approved first line treatment options. Both combinations are approved for all IMDC risk groups. The Pembrolizumab and Axitinib combination demonstrated significant improvement for OS at 12 months versus Sunitinib (89.9% vs. 78.3%) as well as longer median progression free survival (PFS), (15.1 months vs. 11.1 months, HR 0.69; 95% CI, 0.57– 0.84; *p* < 0.001). Similarly, in JAVELIN Renal 101, the Avelumab and Axitinib combination showed better PFS than Sunitinib although no OS advantage has been observed to date [16]. Both trials included more IMDC intermediate- and poor-risk than favorable-risk patients, however sub-group analysis demonstrated clinical benefit across all IMDC risk groups, specifically including the favorable risk cohort. CheckMate 9ER also looked at ICI/TKI combination for all IMDC risk groups and initial results presented at ESMO 2020 favored combination of Nivolumab and Cabozantinib over Sunitinib with manageable toxicities. Median PFS was doubled with combination Nivolumab and Cabozantinib (16.6 months) versus Sunitinib (8.3 months), (HR 0.51; 95% CI, 0.41–0.64; *p* < 0.0001) and OS also favored the Nivolumab/Cabozantinib combination (HR 0.60; 98.89 CI 0.40–0.89); *p* = 0.0010) [17]. There are two other options that have recently been approved for intermediate and poor risk patients; the combination of Nivolumab and Ipilimumab, and single agent Cabozantinib. However, neither has demonstrated efficacy advantages over Sunitinib for favorable risk patients and as such are not considered appropriate options at this time.

Active surveillance is also considered to be an appropriate option for some favorable risk patients if they have asymptomatic low volume disease and the trajectory of the progression is relatively slow [18,19]. Rini et al. performed a prospective phase II trial evaluating active surveillance for advanced RCC and reported PFS of 17.0% and 11.0% for the entire cohort at 24 and 36 months respectively [18]. In this trial, the number of involved organs and IMDC risk factors were independently associated with shorter PFS. This approach is certainly feasible for patients with indolent disease where it can help to preserve quality of life for a longer period of time seemingly without compromising future outcomes. Although with increasing numbers of increasingly effective options, its use is becoming more selective.

In summary, for patients with favorable risk disease who have met a threshold for requiring active systemic anti-cancer therapy, a combination of ICI and TKI is now considered the standard first line regimen where available, given the demonstrated superior efficacy of these combinations over Sunitinib. However single agent TKIs have long-proven efficacy and are still appropriate in a subset of patients, particularly when the risk ICI-induced toxicity is a concern.

ICI have a prominent role to play in the treatment of intermediate- and poor-risk patients based on the published data. Historically, Sunitinib and Pazopanib were widely used in these patients, as was Temsirolimus particularly in the mid 2000s, on the basis of data showing a survival advantage for Temsirolimus over IFN-a in poor risk patients as defined in the trial criteria [20]. However, in the last couple of years those options have been superseded. Current recommended options to treat these subgroups are combinations of Ipilimumab/Nivolumab, Pembrolizumab/Axitinib and Avelumab/Axitinib, or single agent Cabozantinib.

The Ipilimumab and Nivolumab combination had statistically superior efficacy compared to Sunitinib in intermediate- and poor-risk subgroups, most notably for the primary end point of OS. In CheckMate 214, Ipilimumab and Nivolumab produced an objective response rate (ORR) of 42.0% and a striking complete response rate (CR) of 11.0% in intermediate- and poor-risk patients (Table 1) [20,21,22,23,24,25,26]. Updated data presented at ASCO GU 2020 reported median OS of 47 months with Ipilimumab and Nivolumab versus 26.6 months with Sunitinib (HR 0.66; 95% CI 0.55–0.90; *p* < 0.0001) with median follow up of 42.0 months. The durability of benefit was confirmed in this update as median duration of response (DOR) was still not reached for the responders. The Pembrolizumab and Axitinib combination demonstrated a CR rate of 5.8% in Keynote 426. Subgroup analyses showed 12-month OS and PFS of 91.4% and 70.3% in Pembrolizumab and Axitinib arm for intermediate- and poor-risk patients versus 76.7% and 45.2% with Sunitinib respectively.

Cabozantinib is a second generation multi-targeted TKI which was granted accelerated approval by the FDA for the first line therapy for IMDC intermediate- and poor-risk RCC patients based on the CABOSUN trial; a randomized phase II trial compared with Sunitinib [22]. An update of the trial was published in 2018 which confirmed the preliminary results [27]. This trial reported median PFS of 8.6 months with Cabozantinib compared with 5.3 months with Sunitinib (HR 0.48; 95% CI 0.31–0.74; *p* = 0.0008) with one patient achieving CR.

There are no available direct comparisons of the above-mentioned treatment options and trial populations differ between the studies although of note each used the same standard comparator of Sunitinib. The benefits of these newer regimens over Sunitinib, based on the data described, are now reflected in current international guidelines with the exception of the very recently presented data for Nivolumab/Cabozantanib which has not yet been evaluated for approval [13,14]. At present the combination of Ipilimumab and Nivolumab has the longest follow-up and therefore the most mature data for durability of response at this time. In addition, a recent update showed that anti-VEGFr TKIs in the second-line setting post Ipilimumab and Nivolumab do seem to retain their efficacy [28]. The data for Keynote 426 and JAVELIN Renal 101 are not yet as mature; time will demonstrate their effectiveness for long term disease control. Cabozantinib monotherapy does have good efficacy in this setting, however this was a randomized phase II rather than a phase III trial, evidence of durable benefit is lacking, and as such its use should be restricted to situations where ICI-containing regimens are not appropriate or unavailable.

### 2.2. Histology-Based Treatment Selection

Knowledge of the histological sub-type of mRCC can be used to guide treatment. At a patient level, it is determined by sampling the original nephrectomy specimen if available, or from a biopsy taken at the time of relapse or of diagnosis of metastatic disease. ccRCC accounts for over 80.0% of all cases of RCC [2], meaning that the vast majority of data arise from clinical trials conducted in this histological sub-type. Non-ccRCC is recognized as a collection of separate entities, with clinical and morphological characteristics that differ from ccRCC and from each other. This is important because RCCs with differing histologies are associated with distinct genomic alterations, clinical features, and manifestations. It is therefore to be expected that these histologies confer differing responses to and outcomes with available regimens.

Systemic anticancer agents approved for treatment of ccRCC are often used in patients with non-ccRCC. However, the described ORR, durations of response, and OS in patients with non-ccRCC are typically inferior in the literature. However, the non-ccRCC histological sub-types are frequently excluded from larger randomized phase III trials thus most existing data in these groups are derived from fairly small prospective phase II trials or subgroup analyses. In fact, despite collectively representing up to a quarter of all RCC histological subtypes, there are currently no specific FDA and EMA approved treatment options for non-ccRCC alone [29]. This section will focus on the benefit of determining histology to guide treatment, particularly in the non-ccRCC histology subtypes in which appropriate choices are less apparent from guidelines due to their relative rarity and consequent lack of robust randomized trial data.

One trial that did allow inclusion of differing histological sub-types of mRCC was the randomized phase II RECORD-3 trial. This trial compared first-line Sunitinib followed by Everolimus at progression, with first-line Everolimus followed by Sunitinib at progression in patients with metastatic RCC of whom 86% had ccRCC and 14% had non-ccRCC [30]. In the trial as a whole, the results favored use of Sunitinib over Everolimus first-line (PFS of 10.7 vs. 7.9 months; HR 1.43; 95% CI 1.15–1.77). When the ccRCC group was sub-analyzed, although there was no statistical difference in OS between the two arms there was a notable numerical difference. Median OS was 23.9 months for Everolimus followed by Sunitinib (*n =* 207) and 30.2 months for Sunitinib followed by Everolimus (*n* = 197) (HR 1.1; 95% CI 0.9–1.4). Thus, the results favored selecting anti-VEGFr TKIs in preference to mTOR inhibitors in the first line setting in ccRCC. This is reflected in guidelines for treatment of metastatic RCC worldwide. In contrast, the differences in PFS and OS seen in the two different sequences were not replicated in the non-ccRCC cohort. Median OS in the non-ccRCC subgroup was 16.2 months for sequential Everolimus and Sunitinib (*n* = 29) and 16.8 months for sequential Sunitinib and Everolimus (*n* = 35) (HR 1.0; 95% CI 0.6–1.8). Clearly, the OS from both agents was lower in the non-ccRCC cohort but not noticeably different from each other. This lack of observed difference could be in part due to the smaller numbers in this cohort, or it could indicate a much lower responsiveness to Sunitinib and/or that Everolimus does have some activity against non-ccRCC.

The use of anti-VEGFr-TKIs in non-ccRCC has also been investigated in a number of dedicated trials. One small retrospective phase II study of 53 patients did show a benefit for Sunitinib over Sorafenib in PRCC, with an increased PFS (11.9 vs. 5.1 months, *p* < 0.001) (Table 2) [31,32,33,34,35,36,37]. The same study did not show any statistically significant difference in the PFS between Sunitinib and Sorafenib in a small number (*n* = 11) of chromophobe RCC (ChRCC). Similarly, mTOR inhibitors were first studied over a decade ago across all RCC subtypes; the subgroup analysis in the phase III ARCC trial supported the use of Temsirolimus over IFN-α in non-ccRCC, with an improved OS (8.2 vs. 4.3 months; HR 0.49; 95% CI 0.29–0.85). Although this was a retrospective analysis, it led to the option of using mTOR inhibitors in the treatment of non-ccRCC, and subsequently its use as one of the arms in later non-ccRCC trials. Subsequently two notable trials in non-ccRCC were developed that compared Sunitinib and Everolimus. Both showed activity for these drugs, although the efficacy seemed less than that previously seen in ccRCC [38,39]. In the ASPEN Trial, Sunitinib improved PFS compared with Everolimus across the whole cohort of 108 patients Sunitinib (8.3 months 80% CI 5.8–11.4 versus 5.6 months 80% CI 5.5–6.0; HR 1.41 80% CI 1.03–1.92; *p* = 0.16). However, this was not consistent and in the small number of patients (*n* = 16) with ChRCC the PFS numerically favored Everolimus [39]. However, the number of ChRCC patients were too low to reach statistical significance. The randomized phase II ESPN trial similarly demonstrated that both agents had modest efficacy with a small numerical but non-statistically superior efficacy advantage for Sunitinib [38].

The efficacy of immunotherapy-based regimens is also being investigated in non-ccRCC subtypes in both retrospective and prospective studies. One retrospective multicenter analysis of 43 patients with metastatic non-ccRCC who had had a variety of PD-1- or PD-L-1-targeting agents found that ORR, the primary endpoint of the study, was 19.0% in this heterogenous group. This included responses in patients with papillary non-ccRCC and those whose cancers had sarcomatoid differentiation [34]. Two prospective studies have also shown efficacy of immunotherapy in treating non-ccRCC. In the phase IIIb/IV CheckMate 374 study, the ORR in 44 patients treated with Nivolumab was 13.6% at a median follow-up of 11 months [40]. Most subtypes of non-ccRCC were all fairly well-represented in this study, with one patient with ChRCC showing a complete response to Nivolumab. In the single-arm phase II Keynote 427 study which looked at Pembrolizumab as a first-line treatment option, the preliminary data show an ORR of 25.0% for PRCC, 9.5% for ChRCC, and 35.0% for unclassified RCC, with a median follow-up of 11 months [35]. There have been several studies looking at immunotherapy in combination with other agents, such as MET inhibitors, other immunotherapy agents, and VEGF inhibitors. For example, the combination of Durvalumab and Savolitinib was looked at in 42 patients with PRCC, with an ORR of 29.0% [41]. The combination of Nivolumab and Ipilimumab showed an ORR of 28.0% when studied in 18 patients with various types of non-ccRCC [36]. Atezolizumab and Bevacizumab was also studied in 60 patients with various types of RCC, with an ORR of 26.0% across the cohort of non-ccRCC [42].

Renal cell cancers with sarcomatoid differentiation are not recognized as a separate entity (as per the 2016 WHO criteria) but deserve recognition as they are generally associated with a poorer outcome [43]. Immunotherapy has been a treatment of interest in RCC with sarcomatoid differentiation. Research has suggested that sarcomatoid tumors have a high degree of inflammation, and often have poor-risk features, and so may be sensitive to ICI as indicated in the retrospective analysis described above [34]. This has been further supported by three recent prospective trials looking at immunotherapy to treat RCC in ccRCC with sub-analyses focusing on tumors that including sarcomatoid differentiation. The Keynote 426 trial which compared Axitinib/Pembrolizumab to Sunitinib included 105 patients with sarcomatoid features [33]. The results heavily favored the combination treatment arm with PFS not reached, against 8.4 months in the Sunitinib arm (HR 0.54; 95% CI 0.29–1.00), and with an ORR of 58.8%. Similarly, in the ImMotion 151 trial which compared Atezolizumab/Bevacizumab with Sunitinib, there were 142 patients analysed that had tumors with sarcomatoid differentiation [44]. Although this combination is not currently being further developed in RCC, the findings are relevant regarding the efficacy in the sarcomatoid subgroup. There was strong evidence favoring the combination arm, with improved PFS in the intention to treat (ITT) population (HR 0.56; 95% CI 0.38–0.83). An update from the CheckMate 214 study was recently published, looking specifically at subgroup analyses including tumors with sarcomatoid differentiation [45]. The PFS achieved with Ipilimumab/Nivolumab was superior to Sunitinib with HR 0.61 (95% CI 0.38–0.97), further demonstrating the efficacy of immunotherapy combinations in RCC with sarcomatoid differentiation.

Translocation renal cell carcinoma (TRCC) constitutes only 1–4% of adult RCCs and typically responds poorly to conventional ccRCC therapies. It is associated with TFE3, TFEB, or MITF gene fusions, with various associated targetable signaling pathways. An example is the presence of an *SFPQ-TFE* fusion [t(X;1) (p11.2; p34)] resulting in “Xp11.2 translocation carcinoma”, in which TFE3 chromatin immunoprecipitation followed by deep sequencing analysis indicated a strong enrichment for the PI3K/AKT/mTOR pathway [46]. This led to the concept of targeting both the PI3K/AKT and mTOR pathways simultaneously, and while no agent has yet made it to a large-scale clinical trial, there have been some promising results in smaller studies. The novel inhibitor SN202, a dual inhibitor of PI3K and mTOR pathways, has been studied in vitro and in mice, with a decrease in the phosphorylation of PI3K downstream signaling molecules AKT and S6K in renal cancer cells seen [47]. Similarly, miR-205-5p is a negative regulator of both PI3K/AKT and mTOR pathways which has also been shown to decrease the production of renal cancer cells in vitro and in mice, through both direct targeting of vascular endothelial growth factor A and promotion of apoptosis and inhibition of epithelial to mesenchymal transition in renal cancer cells, leading to reduced cell proliferation, invasion and migration of ccRCC [48]. Although at present these tumors are treated according to algorithms for ccRCC or for other non-ccRCC sub-types, it is hoped these results will yield other approaches for possible future therapies for TRCC.

Renal medullary carcinoma (RMC) is an aggressive subtype of RCC, with up to 94% of patients presenting with stage IV disease [49]. RMC makes up <0.5% of all RCCs, and frequently affects young adults with haemoglobinopathies such as sickle cell trait [50]. For many years, the mainstay of treatment has been cytotoxic chemotherapy, most commonly with platinum-containing doublet regimens [51]. However durable benefit is rare and the search for more effective therapeutic strategies is ongoing. Case reports have described responses to ICIs in RMC with one reporting a CR in one of three patients with RMC treated with Nivolumab [51]. Three clinical trials of ICIs alone or in combination are now recruiting in RMC.

Chemotherapy has a place in the treatment of a small group of non-ccRCC, despite a relative paucity of evidence. Oudard et al. conducted an open-label phase II trial of 23 patients with collecting duct RCC where ORR of 26.0% were achieved Cisplatin and Gemcitabine with median OS of approximately 11 months [37]. Conventional RCC-type approaches also lead to responses in some patients with collecting duct RCC. Combining these approaches, it was found that the addition of Bevacizumab to chemotherapy produced improved PFS and OS in collecting duct carcinomas (CDCs) (15.1 and 27.8 months, respectively) [52]. Although this was a small study, the results suggested Bevacizumab plus chemotherapy may be a feasible treatment option in this otherwise morbid tumor subtype.

### 2.3. Toxicity-Driven Treatment Selection and Modification

All delivered treatments confer toxicities that differ both between regimens and patients. Their presence or absence, and severity, can be usefully employed to individualize treatment regimens for patients. There is considerable research effort to identify and utilize biomarkers for toxicity but none are in routine practice in RCC at this time; toxicities are more often used to adjust treatment than to select it. Strategies used to modify treatment in response to toxicity are dose reduction, dose interruption, schedule modification, and regimen change and in all cases should be accompanied by supportive therapies for toxicity management.

Based on considerable experience with Sunitinib in particular, there is now evidence that modified dosing and scheduling of TKIs can be effectively used for treatment individualization. All phase III trials of Sunitinib used the standard, licensed dosing schedule of four weeks on treatment followed by a two week break from treatment (4/2). However, we know that there is inter-patient variability in tolerating the standard schedule (SS). A number of studies have been conducted to compare the standard dosing schedule with altered schedules (AS) to evaluate whether AS could deliver improved tolerability whilst retaining drug efficacy. RESTORE was a prospective phase II randomized trial which compared 4/2 schedule with two weeks on, one week off (2/1) schedule. In this trial, failure free survival (FFS) was defined as treatment discontinuation due to disease progression or treatment toxicities. The alternate “two weeks on, one week off” (2/1) Sunitinib schedule was associated with 63.0% FFS at 6 months as compared to 44.0% with the standard 4/2 schedule. Median time to treatment failure (TTF) was also statistically better with 7.6 months for the 2/1 schedule versus 6.0 months for 4/2 schedule (*p* = 0.029) [23]. Another prospective phase II trial evaluated the efficacy and safety of Sunitinib in the first line setting utilizing an individualized approach. Patients had the dose of Sunitinib, and the number of days on treatment, adjusted according to reported rates of toxicity with an aim that toxicities remained ≤ grade II intensity. This trial reported PFS of 12.5 months (*p* < 0.001) and clinical benefit rate of 84.6% with the individualized approached [24]. A number of retrospective analyses have also shown that alternate dosing schedules, particularly the 2/1 schedule, can deliver good efficacy with acceptable tolerability [53,54]. Whilst the design of these studies varies, and they were not powered to demonstrate superiority of AS for efficacy, collectively they suggest that Sunitinib schedule and the dose can be personalized according to adverse effects without losing its efficacy.

The earlier generation VEGFr-TKIs differ slightly in their side effect profiles but have broadly similar and overlapping toxicities. However interestingly, individual patients can develop different side effects to each of these TKIs therefore switching between TKIs to another can improve tolerability whilst retaining efficacy and cancer control. PISCES was a phase IIIb randomized, double blind, cross-over trial evaluating patients’ preference for Pazopanib versus Sunitinib. Patient preference was assessed by questionnaire at the end of the two treatment periods and the results overall favored Pazopanib but not in all patents (Table 1) [25]. Therefore, adjusting on an individual patient basis and being prepared to switch regimen is an important principle that remains relevant despite these treatments themselves now being largely superseded.

While the data for the altered dosing of Sunitinib comes from phase II trials and retrospective studies, Axitinib dose escalation and de-escalation strategies have been evaluated prospectively in phase III trials. In a pharmacokinetic and pharmacodynamic study of Axitinib, a clear association was found between circulating drug levels and efficacy of Axitinib; higher ORR was seen with higher drug exposure [55]. The same study demonstrated that the presence of Axitinib-induced hypertension, an “on-target” toxicity, could be used to guide Axitinib dose in order to optimize efficacy. It showed that amongst patients who developed diastolic blood pressure (dBP) of ≥90 mmHg in response to Axitinib median PFS was 14.6 months as compared to 7.8 months in the cohort in whom dBP remained <90 mmHg whilst on Axitinib.

Patients develop specific toxicities from ICI known as immune-related adverse events. However, there is overlap with those toxicities caused by TKIs, e.g., diarrhea, elevated liver enzymes, and skin rashes. The emergence of ICI and TKI in combination as a treatment strategy, poses a new challenge to identify the cause of emergent toxicities and to adjust treatment accordingly. The consensus statement from The Society for Immunotherapy of Cancer, described differing possible approaches for overlapping toxicities. The majority of the committee recommended stopping the TKI for 2–3 days as a first step given the shorter half-life of TKIs, particularly Axitinib, which is used as a backbone in two of these regimens. Others recommended holding both drugs with or without steroids, or continuing Axitinib but holding ICI and treat with steroids [56]. Ultimately the choice depends on the specific toxicity, its grade and physician judgement.

### 2.4. Consideration of Comorbidities in Treatment Selection

As with any disease, patients with more significant comorbidities and worse performance status may have a poorer outcome than those with fewer comorbidities and good performance status. The effect of comorbidities (including age) were studied in a retrospective analysis, with data coming from the Surveillance Epidemiology and End Results (SEER) database in the United States [57]. The Charlson Comorbidity Index (CCI) was used, with a higher score indicating the presence of more severe comorbidities. It was found that patients with a higher CCI had decreasing probabilities of receiving systemic treatment versus no treatment, when all patients with RCC were studied (OR 0.86; 95% CI 0.77–0.96). For all patients who were alive at the landmark six month point of analysis, there was a statistically non-significant swing towards those who had received some form of systemic treatment (OR 1.04; 95% CI 0.87–1.23). This could be explained by the fact that those who are alive at six months were more likely to have a better performance status and/or higher pre-treatment estimate of longer OS, thus were more suitable for active therapy. Also, a study looking at comorbidities as prognostic markers showed that while the investigated co-morbidities could induce pathophysiological changes that predisposed to tumor progression, none were independent prognostic factors in patients with RCC [58].

It is often appropriate for severely comorbid patients with advanced RCC not to receive any systemic anti-cancer therapy, with more of a focus on symptomatic relief and improved quality of life. However active, treatment should be considered in stage IV RCC and can be deliverable to many patients by adapting choices with knowledge of their specific comorbidities. This is especially relevant in the present paradigm, given the range of systemic treatment options with manageable tolerability [59]. One example is selecting immunotherapy or mTOR inhibitors in patients with advanced RCC who have existing significant circulatory disease, to avoid VEGFr-TKI-driven increase in the risk of vascular events. Conversely, patients who have inflammatory-driven pathologies (e.g., inflammatory bowel disease, autoimmune diseases) are at higher risk of adverse effects from immunotherapy and according to the severity, may be more suited to a targeted therapy option. Although there are few situations where individual therapies are absolutely contraindicated, the knowledge of comorbidities and expected toxicities can guide preferences to limit the risk of exacerbating established comorbidities.

### 2.5. Assessment of Disease Distribution to Guide Treatment

Consideration of the sites and extent of RCC metastases can also be helpful in driving treatment selection. For example, it has long been standard practice to utilize local treatment modalities for patients with a limited metastatic burden. In such cases, surgical resection (metastasectomy) and/or stereotactic radiosurgery can lead to excellent disease control whilst avoiding the duration of exposure to, and toxicities from, systemic anticancer treatments [60,61]. In addition, the location of metastases and overall tumor burden, can also have relevance for individualizing treatment.

One example is the choice of initial surveillance for a subset of RCC patients with low metastatic burden that is not amenable to a local therapy option [18]. A real-world study showed that stage IV RCC patients with lung (HR 1.27; 95% CI 1.06–1.53), liver (HR 1.42; 95% CI 1.10–1.84), and bone (HR 1.37; 95% CI 1.13–1.66) metastases respectively had shorter OS than those without these sites of disease [62]. Other studies have analyzed the effectiveness of treatment regimens according to locations of metastases. A sub-analysis of the METEOR trial focused on outcomes in patients with bone metastases. This showed that for patients with bone metastases at baseline, treatment with Cabozantinib versus Everolimus, led to a significant improvement in PFS (7.4 months vs. 2.7 months; HR 0.33, 95% CI 0.21–0.51), OS (20.1 months v 12.1 months; HR 0.54, 95% CI 0.34–0.84) and ORR (17.0% vs. 0%), as well as improved response on bone scan (20.0% vs. 10.0%) [63]. The CABOSUN trial also demonstrated that the degree of benefit for Cabozantinib over Sunitinib was greater for patients with metastatic bone disease. The median PFS in patients with bone disease was almost double with 6.1 months for Cabozantinib as compared to 3.3 months for Sunitinib with an impressive HR of 0.54 [22]. Cabozantinib has become widely recognized as an appropriate TKI for RCC patients with bone metastases.

A further example is the observation that the presence of metastases to endocrine (or “glandular”) organs is associated with relatively favorable outcomes in patients with advanced ccRCC [26]. At first presentation of metastatic RCC, patients with at least one glandular metastasis compared to those without glandular metastases were more likely to have favorable risk disease (37.2% vs. 18.0%), less likely to have poor-risk disease (10.7% vs. 27.0%), and had considerably longer OS (61.5 months vs. 37.4 months, HR 1.70; 95% CI 1.30–2.20, *p* < 0.001) (Table 1). Interestingly, these findings were irrespective of the presence or absence of bone or liver metastases, suggesting that the potential for improved OS must be recognized when determining management of patients with advanced ccRCC and glandular metastases. This is relevant, as it may affect treatment choice in this subgroup, particularly in those with limited sites of metastatic disease in whom a radical treatment approach should be adopted where possible. If a radical approach is not deemed possible, it is reasonable to adopt a period of initial surveillance to avoid the toxicities of systemic therapy, as the disease may remain indolent for a long period of time and potentially for a number of years.

Conversely, brain metastases confer a relatively poor prognosis, which is often less than 12 months from the time of diagnosis [64]. While this is frequently still the case, patients are receiving more aggressive intervention for brain metastases such as the increased use of stereotactic brain radiosurgery. This has led to improved local control of intracranial metastases particularly for those with a solitary brain deposits. For example, Suarez-Sarmiento Jr et al. showed that relapse free survival (RFS) in RCC patients with brain metastases was correlated with the number of brain lesions; RFS was 27.5 months for those with only one lesion, but 12 months for those with more than one lesion (*p* = 0.0026) [64]. This supports a more aggressive approach to limited brain metastases in patients with RCC.

A further factor that may influence treatment selection is whether there is any clinical benefit in achieving tumor shrinkage, as opposed to stability, in order to improve cancer-related symptoms. This is particularly relevant in anatomical sites where tumor growth may lead to adverse consequences, such as metastases affecting the spinal canal, and mediastinum disease. In such cases, the likelihood of inducing meaningful disease response, and the time to achieve response can be relevant in guiding therapy choice. Regimens that have achieved relatively short time to treatment response (TTR), in addition to meeting their survival endpoints, include Nivolumab plus Ipilimumab in CheckMate 214, Pembrolizumab plus Axitinib in KEYNOTE 426 and most recently Nivolumab plus Cabozantanib in Checkmate 9-ER. In these trials, ORR with Nivolumab plus Ipilimumab was 42%, and was 56% with both Pembrolizumab plus Axitinib and Nivolumab plus Cabozantanib. It is also worth noting that the progressive disease rate was higher with Nivolumab plus Ipilimumab at 27%, compared to 14.6% for Pembrolizumab plus Axitinib and 13.7% with Nivolumab plus Cabozantanib. Acknowledging the limitations of cross trial comparisons, this suggests that in cases where a rapid response is desirable, a reasonable strategy is to select a TKI–containing regimen, ideally as a TKI + ICI combination.

### 2.6. Role of Genomic Markers and Biomarkers in Treatment Selection

Potential benefits of comprehensive genomic analyses of renal cancers include the identification of biomarkers for prognosis and/or prediction of treatment benefit, and in identifying pathways suitable for targeted therapies. Each subtype of RCC has a unique pathophysiology that may be appropriate for separate therapeutic development [29,65]. Furthermore, many genetic/familial syndromes are strongly associated with specific gene mutations and the presence of RCC subtypes. Numerous active therapeutic trials have been designed in utilizing this paradigm, evaluating treatments in both ccRCC and non-ccRCC.

ccRCC is characterized by the inactivation of Von Hippel-Lindau (VHL) tumor suppressor protein and subsequent VHL mutation in essentially all cases, which results in the accumulation of hypoxia-inducible factor (HIF) and subsequent downstream activation of pathways involved in cell metabolism, proliferation and angiogenesis. Several ongoing studies are addressing the development of HIF-targeted therapeutic approaches. The CXC-chemokine receptor-4 (CXCR4) inhibitor X4P-001, which has been shown to downregulate HIF-2α, is being studied in combination with Axitinib, with the intent of overcoming or delaying resistance to VEGFr TKIs (Table 3) [66,67,68,69,70,71,72,73,74,75,76,77,78,79]. PT2385 is a direct HIF-2α antagonist that is being evaluated in patients with ccRCC in a world-first study [67], with the preliminary results showing a favorable safety profile and activity in patients with heavily pre-treated ccRCC. Recently the HIF-2α antagonist MK-6482 was studied in a small group of patients with ccRCC, with PFS at 12 months of 98% (95% CI 89–100%), and DOR) in confirmed responders not reached (range 12–62 weeks) [68].

In patients with ccRCC, PBRM1 is the second most commonly altered gene, with up to 40% of these cancers having somatic loss-of-function mutations [50]. In the localized disease setting, loss of PBRM1 is associated with unfavorable clinical outcomes, with these patients more likely to have stage III disease at presentation, as well as more aggressive pathology, and a higher likelihood of developing stage IV disease in the future [69]. There have been other histone modifying and chromatin remodeling genes in ccRCC studied (SETD2, BAP1, KDM5C), all of which are associated with advanced stage, grade, and possibly worse cancer-specific survival [69]. In the metastatic setting, however, loss of PBRM1 is associated with both improved PFS and OS [70], and may be explained by tumors harboring PBRM1 mutations being strongly angiogenic, resulting in the upregulation of targets of VEGF-directed therapies (e.g., HIF) [80]. The favorable outcome associated with loss of PBRM1 is also found in the RECORD-3 and ImMotion150 trials, irrespective of treatment choice in each trial [71,72].

PRCC has a long list of associated genetic mutations [81]. Type 1 PRCC has been associated with both alterations in the MET gene and a gain in chromosome 7 (where the MET gene is located). There have been some promising results in recent studies looking at treatment of type 1 PRCC with MET inhibitors. Crizotinib is a small-molecule TKI that inhibits MET as well as anaplastic lymphoma kinase (ALK) and ROS proto-oncogene 1 receptor tyrosine kinase (ROS1), which has been shown to be effective in Type 1 PRCC. The effect of Crizotinib in type 1 PRCC patients was studied in tumors with MET-positivity and/or MET-amplification, against those that were MET-negative and not MET-amplified [73]. PFS at one year was 80.0% (95% CI 20.4–96.9) in the MET-positive/amplified arm, against 22.0% (95% CI 5.4–45.6) in the MET-negative/non-amplified arm. The OS was relatively similar in each arm, and the numbers in this study were quite small (23 evaluable subjects), but nevertheless it may provide us with a potential treatment option for type 1 PRCC and is currently being trialed in larger studies. Furthermore, two respective phase II trials looking at Foretinib and Savolitinib, both multi-kinase inhibitors targeting MET, demonstrated significant response rates in patients with MET-driven papillary RCC’s [82,83]. Foretinib had a 50.0% response rate among patients with a germline MET mutation, and Savolitinib had a 18.0% ORR in MET-driven tumors (compared to 0% ORR in non-MET driven tumors) with a median PFS of 6.2 months against 1.4 months in the respective arms (HR 0.33, 95% CI 0.20–0.52). There is an ongoing phase II randomized controlled trial which was designed to compare Crizotinib, Savolitinib, Cabozantinib, and Sunitinib in patients with PRCC [84]. The Crizotinib and Savolitinib arms were closed in mid-2018 for futility after an interim analysis, leaving the efficacy of Cabozantinib and Sunitinib to be studied in both MET mutated and MET expressing tumors. However, none of the trials has yet yielded results that translate into mutation-driven selection in clinical practice. At present, appropriate first line choices for MET-positive non-ccRCC on a biological basis could be Sunitinib or Cabozantinib. Cabozantinib has activity against MET as well as other signaling pathways but lacks clinical trial evidence in phase III clinical trials in non-ccRCC. Sunitinib, conversely, is listed as the default option in many clinical guidelines, based on the trial results described previously. Type 2 PRCC has been linked to mutations in CDKN2A, SETD2, BAP1, PBRM1, TERT, NF2, FH, and NRF2-ARE pathway genes (among others), as well as a CpG island methylator phenotype [85]. Various mutations in CDK2NA have also been seen frequently in CDCs although it is unclear whether and how this knowledge may be harnessed in future therapy selection [86].

The chromophobic and oncocytic subtypes have a correlation with Birt-Hogg-Dubé syndrome. Various types of non-ccRCC have been associated with Tuberous Sclerosis Complex (TSC) with one study showing a high incidence of both papillary and chromophobe/oncocytic subtypes [87]. Apart from MET-mutated PRCC, there is no strong evidence for other mutations due to a lack of adequately sized cohorts, although the trials are ongoing. As an example, given the known association, the presence of FH may lead to the use of treatment regimens efficacious in patients with hereditary leiomyomata and renal cell cancer (HLRCC) or PRCC [50].

An association between mutation status for TSC1/TSC2/mTOR and therapeutic outcome with Everolimus was tested, but not confirmed. Clinically meaningful differences in PFS, however, were seen based on PTEN expression by IHC, which was lost in >50% of patients [74]. A loss of PTEN IHC expression led to favorable outcomes in patients treated with Everolimus when compared to retained PTEN IHC expression (median PFS 10.5 vs. 5.3 months, HR 2.5, *p* < 0.001). Such differences were not seen with Sunitinib, suggesting that TKIs may be a preferred therapeutic option in those with retained PTEN IHC expression.

For ICI across all tumor types as well as in RCC, PD-L1 expression is the most thoroughly researched biomarker. Its expression has an association with aggressive disease biology in RCC, including high nuclear grade, lymph node involvement, and distant metastases; however, its utility as a predictive biomarker is limited as it lacks negative predictive value [75,76]. Though treatment efficacy is greater in PD-L1 positive patients, it has been repeatedly demonstrated that ICI also provide clinical benefit to PD-L1 negative patients [16,33,44]. Hence, PD-L1 expression does not help segregate responders from non-responders. The proposed explanations of this inconsistency as a biomarker are variable expression of PD-L1 on fresh versus archival tissue, intra-tumoral heterogeneity, and heterogenous expression of PD-L1 on primary versus metastatic sites, as well as lack of using a uniform assay [88]. Interestingly however, an association with high PD-L1 and poor outcome when treated with VEGFr inhibitors has been shown [77].

Tumor mutational burden (TMB) is perhaps the most promising biomarker for ICI in recent times for tumor types with high mutational loads such as melanoma and urothelial cancer. However, the mutational load in RCC is usually very low and as yet, it is not in routine clinical use as a predictive biomarker in any setting. The generation of tumor neoantigens may come from higher frequencies of frameshift insertion and deletion mutations [89], and the ongoing TRACERx Renal study has shown secondary mutations and chromosomal changes involved in tumor evolution, outlining their clinical relevance [90]. In the IMmotion 150 trial, exploratory biomarker analysis was conducted evaluating TMB and neo-antigen burden but found no association with PFS [72]. Strongly angiogenic tumors are more responsive to anti-VEGF TKI as compared to ICI. Gene expression profiling can identify RCC into high and low angiogenic tumors. As mentioned previously, tumors harboring PBRM1 mutations tend to be strongly angiogenic while BAP1 mutation is associated with poorly angiogenic tumors. These genetic signatures were integrated prospectively in phase III study (IMmotion 151), with the data recently validating both angiogenesis and T-effector gene signatures as predictors of outcome [44,90]. Patients with favorable risk RCC are characterized by an angiogenesis^High^ gene signature. In renal tumors with T-effector^High^ and angiogenesis^Low^ signatures, Atezolizumab/Bevacizumab improved PFS compared to Sunitinib. Patients with an angiogenesis^High^ gene signature had improved PFS compared to angiogenesis^Low^ group in the Sunitinib arm. Supporting the data mentioned previously on tumors with sarcomatoid differentiation, sarcomatoid RCCs were shown to be characterized by angiogenesis^Low^ and T-effector^High^ gene signatures, with higher PD-L1 expression, and hence greater benefit with immunotherapy [78]. This may be a significant step in the understanding of the biology of RCC, and the subsequent information obtained to direct therapy.

Circulating tumor DNA (ctDNA) is the most widely used blood-based test for assessment of cancer biomarkers. It is a means of assessing for tumor-based material less invasively, and its use as a predictive and prognostic biomarker has been validated across multiple solid tumor types [91,92]. The use of ctDNA in RCC is still under investigation, although the rationale is very much justified. Multiple genomic alterations including those in VHL, TP53, EGFR, and NF1 have been identified in ctDNA, with the same report showing genomic alterations in any gene detected in 78.6% of patients with metastatic RCC [79]. The ctDNA count to assess tumor response to treatment has been studied since, with a positive correlation between detectable ctDNA and radiographic burden of disease found [93]. Although the limitations of many studies include a lack of power (due to small sample sizes), more specific genomic analyses would likely benefit further from ctDNA analysis in terms of prognostication, an example being MET-deficient RCC [94].

A new area of focus these days is gut microbiota where pre-clinical studies have shown that certain micro-organisms may predict positive or negative responses from ICI [95]. Exposure to antibiotics and dietary habits impact our gut microbiome and further research is being carried out to segregate the groups of micro-organisms which are consistent with ICI related outcomes.

The available published data for predictive biomarkers is not sufficiently strong to change current clinical practice. Most of the literature is based on retrospective analysis on archival tissues but there are many ongoing studies which are prospectively evaluating various biomarkers. The current evidence to use biomarkers in treatment decision is lacking and needs validation before it can be used for clinical decision making.

## 3. Selection of Second-Line Treatment and beyond

The treatment landscape beyond the first line setting has changed considerably in the last few years, and has been further complicated since several agents previously used in the second and third line settings are now indicated first line, either alone or in combination. In fact, only five years ago the standard of care for second-line treatment was limited to Axitinib or Everolimus for patients who had received a TKI as their first-line treatment for metastatic RCC [96].

The same principles apply in choosing subsequent lines of treatment in advanced RCC to those adopted in choosing initial therapy. There is evidence that patients exposed to ICI upfront can respond well to VEGF-targeting TKIs on progression [28]. This was reported in a follow-on observational study in 33 patients who had progressed on upfront ICI therapy in the Checkmate 214 trial and who subsequently received a VEGFr-TKI. The reported ORR was 36.0% with a median PFS of 7.0–8.0 months achieved with the VEGFr-TKIs Sunitinib, Pazopanib, Cabozantinib, and Axitinib. This was particularly respectable given that the trial population had predominantly intermediate and poor risk disease. These four VEGFr-TKIs together with Tivozanib, and Sorafenib can all be appropriately considered after first-line VEGFr-TKIs on the basis of randomized trial data [97,98,99,100,101,102,103,104,105,106,107,108,109].

There is continuing research into the benefit of immunotherapy in advanced RCC in second and later lines of therapy after a variety of initial treatments. The phase III CheckMate 025 study evaluated efficacy outcomes from Nivolumab and Everolimus in patients who had progressed after one or two prior TKIs. It demonstrated a statistically significant improvement in ORR, PFS, and OS with Nivolumab, compared to Everolimus [12]. No other checkpoint inhibitor therapy agent is yet indicated for use in second-line treatment and beyond in advanced ccRCC although other trials are recruiting including using different ICI-containing regimens in those exposed to upfront ICI therapy.

It has long been known that mTOR inhibitors are also beneficial in treating advanced RCC beyond first-line therapy. Everolimus showed an improved PFS when compared to placebo [110]. With the advance of TKI treatment options, and subsequent randomized trial data showing superiority of both Nivolumab and Cabozantinib over Everolimus, mTOR inhibitors are less-frequently used as monotherapy if other options are available [12,101]. However, the use of Everolimus in combination with the VEGFr-TKI Lenvatinib is approved and in routine use. In a randomized phase II study of 150 patients, the combination of Lenvatinib and Everolimus showed PFS and OS benefit over Everolimus monotherapy [111].

Therapy with IL-2 is now uncommon for treatment of ccRCC. It is FDA-approved for treatment of metastatic RCC but is only available at select institutions world-wide for patients with low-volume disease. ORR are 14.0–20.0%, but patients who have a complete response may enter a long-term remission [6,112]. Toxicity may be profound, and treatment-associated deaths have been reported. Its use has largely been superseded by the advent of the plethora of ICI-based and other treatment regimens.

Selecting the most suitable treatment option for second-line and beyond in advanced RCC is heavily dependent on the regimen given in the first-line setting, in addition to consideration of those factors used to select first line treatment such as efficacy data, local availability, comorbidities, physician/patient preference, and, to a lesser extent, histological subtype. Despite the many therapeutic advancements made in the last few years, there is a lack of well-validated prognostic and predictive biomarkers to further personalize the management of advanced RCC, although this means a very active research field. Based on the efficacy and strength of data, the most widely recommended second-line treatment options for advanced ccRCC are Cabozantinib and Nivolumab although Lenvatinib with Everolimus of merit and is increasingly used [12,100,101]. Beyond this, the options include any of the other approved treatments and of course recruitment to clinical trials is to be encouraged where available.

## 4. Conclusions

Over the last decade, there has been a major shift in the treatment of advanced RCC. The progress of medicine has led to improved survival, making the management of complex disease processes as important as ever. The evolution of both immunotherapy and targeted therapy treatment options, along with the significant advances in research into personalized medicine, have led to greatly improved survival and quality of life in this previously morbid tumor group. Treatment choice is primarily selected utilizing internationally-recognized, evidence-based consensus guidelines. However, choice between treatments that are regarded to have broadly equivalent efficacy is often required and, to date, has been driven by clinico-pathological features including RCC risk category, histological sub-type, patient fitness, and comorbidities. However, the development of these treatment options has been accompanied by enormous advances in understanding of the molecular and genomic basis of RCC. It is therefore hoped that the continuing development of validated tumor genomic markers and biomarkers, together with new ways of predicting and prognosticating outcome, will eventually allow for more educated, accurate and tailored treatment individualization.

## Figures and Tables

**Table 1:** Summary of studies evaluating individualized treatment based on clinicopathological characteristics.

Study	Study Type	Treatment Individualization	Treatment	Treatment Setting	Outcomes
Hudes et al. [20]	Prospective, randomized phase III	Clinical risk category based (Trial protocol defined poor risk group RCC)	Temsirolimus vs. IFN-a vs. Temsirolimus + IFN-a	First line	OS 10.9 vs. 7.3 vs. 8.4 months (*p* = 0.008)
Checkmate 214 [21]	Prospective, randomized phase III	Clinical risk category based (IMDC intermediate- and poor-risk groups RCC)	Nivolumab + Ipilimumab vs. Sunitinib	First line	OS of 47.0 vs. 26.6 months (*p* < 0.0001)
CABOSUN [22]	Prospective, randomized phase II	Clinical risk category based (IMDC intermediate- and poor-risk groups RCC)	Cabozantinib vs. Sunitinib	First line	PFS of 8.6 vs. 5.3 months (*p* = 0.0008)
RESTORE [23]	Prospective, randomized phase II	Toxicity based	Standard Sunitinib schedule (4/2) vs. altered Sunitinib schedule (2/1)	First line TKI	6-month FFS 44.0% vs. 63.0% (*p* = 0.029)
Bjarnason et al. [24]	Prospective phase II	Toxicity based	Individualized Sunitinib dose and scheduled	First line	PFS 12.5 months (*p* < 0.001)
PISCES [25]	Prospective, randomized phase II	Patient preference based	Pazopanib vs. Sunitinib	First line	70% of patients favoured Pazopanib
Gravis et al. [26]	Retrospective	Disease distribution based (Glandular metastasis vs. Non-glandular metastasis at first presentation)	Any systemic treatment	First line and beyond	OS 61.5 vs. 37.4 months (*p* < 0.001)

Abbreviations: IMDC, International Metastatic RCC Database Consortium; OS, overall survival; PFS, progression free survival.

**Table 2:** Select list of studies for non-clear cell renal cell cancer (non-ccRCC) histologies.

Study	Study Type	Histology	Treatment	Outcomes
Choueiri et al [31]	Retrospective analysis	PRCC	Sunitinib vs. Sorafenib	PFS of 11.9 vs. 5.1 months (*p* < 0.001)
Dutcher et al. [32]	Retrospective analysis	Non-ccRCC	Temsirolimus vs. IFN-a	OS of 11.6 vs. 4.3 months (HR 0.49; 95% CI 0.29–0.85)
KEYNOTE 426 [33]	Prospective, randomized phase III (sub-group analysis)	RCC with sarcomatoid differentiation	Pembrolizumab + Axitinib vs. Sunitinib	PFS not reached vs. 8.4 months (HR 0.54; 95% CI 0.29–1.00)
McKay et al. [34]	Retrospective analysis	Non-ccRCC	Monotherapy or combination PD-1/PD-L1 inhibitor	ORR 28% for PRCC 33% for translocation 43% for sarcomatoid/rhabdoid differentiation
KEYNOTE 427 [35]	Prospective, open-label phase II	Non-ccRCC	Pembrolizumab	ORR 25.4% for PRCC 9.5% for ChRCC 34.6% for unclassified RCC
CheckMate 214 [36]	Prospective, randomized phase III (post-hoc analyses)	RCC with sarcomatoid differentiation	Ipilimumab + Nivolumab vs. Sunitinib	ORR of 56.7% vs. 19.2%
Oudard et al. [37]	Prospective, open-label phase II	CDCs	Cisplatin and Gemcitabine	ORR 26.0% OS of 11.0 months

Abbreviations: CDCs, collecting duct carcinomas; ChRCC, chromophobe renal cell cancers; non-ccRCC, non-clear cell renal cell cancer; ORR, objective response rate; OS, overall survival; PFS, progression free survival; PRCC, papillary renal cell cancer; RCC, renal cell cancer.

**Table 3:** Summary of studies using mutations and biomarkers to individualize treatment.

Study	Tumour Subtype	Study Type	Marker	Results, Comments
Atkins et al. [66]	ccRCC	Phase I/II study	HIF2α	CXCR4 + Axitinib delaying ± overcoming resistance to VEGFr inhibitors.
Courtney et al. [67]	ccRCC	Phase I/II study	HIF-2α	PT2385 shows favourable safety profile and activity in patients with heavily pre-treated ccRCC.
Srinivasan et al. [68]	ccRCC	Phase II study	HIF-2α	Improved PFS (98% at 12 months); DOR in confirmed responses NR (range 12–62 months).
Hakimi et al. [69]	ccRCC	Single institution cohort study	PBRM1, SETD2, BAP1, KDM5C	Mutations in all genes are asssociated with advanced stage, grade, and possibly worse CSS
Voss et al. [70]	Advanced/ metastatic RCC	Retrospective cohort study (COMPARZ and RECORD-3)	PBRM1, BAP1, TP53	Loss of PBRM1, gain of BAP1 and/or TP53 associated with improved PFS and OS in stage IV setting
Hsieh et al. [71]	ccRCC	Retrospective analysis (RECORD-3)	PBRM1, BAP1, KDM5C	PBRM1 and BAP1 mutations associated with longer and shorter PFS with 1L Everolimus in stage IV setting; KDM5C mutation assocaited with longer PFS with 1L Sunitinib.
IMmotion 150 [72]	Treatment-naïve stage IV RCC	Randomized, phase II study	TMB, angiogenic gene signature	TMB and neoantigen burden not associated with PFS; angiogenesis and T-effector response strongly associated with PFS
CREATE [73]	Type 1 PRCC	Multicentre, non-randomized, open-label phase II study	MET	Crizotinib improved PFS in MET positive/ amplified arm compared to MET negative/ non-amplified arm (80% v 22%); OS similar in both arms.
Voss et al. [74]	Advanced/ metastatic RCC	Randomized, phase II study	PTEN	Loss of PTEN IHC expression had improved PFS when treated with Everolimus, compared to retained PTEN IHC expression (10.5 months v 5.3 months).
Iacovelli et al. [75] Thompson et al. [76]	Advanced/ metastatic RCC	Systematic review / meta-analysis	PD-L1	Limited utility of PD-L1 as a predictive biomarker due to the lack of negative predictive value.
Choueiri et al. [77]	Metastatic ccRCC	Randomized, phase III study (COMPARZ)	PD-L1	Increased PD-L1 was associated with shorter survival in patients with metastatic RCC receiving VEGFr inhibitor agents.
IMmotion 151 [78]	Treatment-naïve stage IV RCC	Randomized, phase III study	Angiogenic gene signature, T-effector gene signature	Confirmation of angiogenesis and T-effector response strongly associating with PFS; also finding associations between angiogenesis and T-effector response, tissue subtypes, and treatment options.
Pal et al. [79]	Stage IV RCC	Retrospective analysis	ctDNA: TP53, VHL, NF1, EGFR, PIK3CA, ARID1A	Disparity in genomic alteration frequencies in post first-line vs. first-line were in *TP53* (49% vs. 24%), *VHL* (29% vs. 18%), *NF1* (20% vs. 3%), *EGFR* (15% vs. 8%), and *PIK3CA* (17% vs. 8%)

Abbreviations: 1L, first line; ctDNA, circulating tumor DNA; ccRCC, clear cell renal cell cancer; CSS, cancer specific survival; CXCR4, CXC-chemokine receptor-4; DOR, duration of response; HIF-2a, hypoxia-inducible factor-2a; NR, not reported; OS, overall survival; PFS, progression free survival; PD-L1, programmed death-ligand 1; TMB, tumor mutational burden; VEGFr, vascular endothelial growth factor receptor.

## Abbreviations

ALK	anaplastic lymphoma kinase
CCI	Charlson comorbidity index
ccRCC	clear cell renal cell cancer
CDCs	collecting duct carcinomas
ChRCC	chromophobe renal cell cancer
CR	complete response
ctDNA	circulating tumor DNA
CXCR4	CXC-chemokine receptor-4
dBP	diastolic blood pressure
DOR	duration of response
EMA	European medical agency
ESMO	European society for medical oncology
FDA	food and drug administration
FFS	failure free survival
HIF	hypoxia-inducible factor
HLRCC	hereditary leiomyomata and renal cell cancer
ICI	immune checkpoint inhibitors
IFN	interferon
IL-2	interleukin-2
IMDC	International Metastatic RCC Database Consortium
ITT	intention to treat
NCCN	national comprehensive cancer network
ORR	objective response rate
OS	overall survival
PD-1	programmed cell death-1
PD-L1	programmed cell death-ligand 1
PFS	progression free survival
PRCC	papillary renal cell cancer
RCC	renal cell cancer
RFS	relapse free survival;
RMC	renal medullary carcinoma
ROS1, ROS	proto-oncogene 1 receptor tyrosine kinase
SEER	surveillance epidemiology and end results
TKIs	tyrosine kinase inhibitors
TMB	tumor mutational burden
TRCC	translocation renal cell carcinoma
TSC	tuberous sclerosis complex
TTF	time to treatment failure
TTR	time to treatment response
VEGFr	vascular endothelial growth factor receptor
VHL	Von Hippel-Lindau
